# County-Level Social Vulnerability and Emergency Department Visits for Firearm Injuries — 10 U.S. Jurisdictions, January 1, 2018–December 31, 2021

**DOI:** 10.15585/mmwr.mm7127a1

**Published:** 2022-07-08

**Authors:** Miriam E. Van Dyke, May S. Chen, Michael Sheppard, J. Danielle Sharpe, Lakshmi Radhakrishnan, Linda L. Dahlberg, Thomas R. Simon, Marissa L. Zwald

**Affiliations:** ^1^Epidemic Intelligence Service, CDC; ^2^National Center for Injury Prevention and Control, CDC; ^3^Center for Surveillance, Epidemiology, and Laboratory Services, CDC; ^4^Geospatial Research, Analysis, and Services Program, Agency for Toxic Substances and Disease Registry, Atlanta, Georgia.

At least 100,000 persons in the United States experience a fatal or nonfatal firearm injury each year.* CDC examined rates of firearm injury emergency department (ED) visits by community social vulnerability using data from CDC’s Firearm Injury Surveillance Through Emergency Rooms (FASTER) program.^†^ ED visit data, shared with CDC’s National Syndromic Surveillance Program (NSSP)^§^ during 2018–2021, were analyzed for 647 counties in 10 FASTER-funded jurisdictions.^¶^ County-level social vulnerability data were obtained from the 2018 Social Vulnerability Index (SVI).** Rates of ED visits for firearm injuries (number of firearm injury ED visits per 100,000 ED visits) were calculated across tertile levels of social vulnerability. Negative binomial regression models were used to estimate rate ratios (RRs) and associated 95% CIs comparing rates of ED visits across social vulnerability levels. During 2018–2021, compared with rates in counties with low overall social vulnerability, the firearm injury ED visit rate was 1.34 times as high in counties with medium social vulnerability and 1.80 times as high in counties with high social vulnerability. Similar patterns were observed for the SVI themes of socioeconomic status and housing type and transportation, but not for the themes of household composition and disability status or racial and ethnic minority status and language proficiency. More timely data^††^ on firearm injury ED visits by social vulnerability can help identify communities disproportionately experiencing elevated firearm injury rates. States and communities can use the best available evidence to implement comprehensive prevention strategies that address inequities in the social and structural conditions that contribute to risk for violence, including creating protective community environments, strengthening economic supports, and intervening to reduce harms and prevent future risk (e.g., with hospital-based violence intervention programs) (*1*,*2*).

In 2021, CDC’s FASTER program was established to provide more timely and comprehensive data on firearm injuries at the state and local levels than were available through traditional data sources. CDC analyzed ED visit data during January 1, 2018–December 31, 2021, for 647 counties in 10 FASTER-funded jurisdictions. Aggregated data were shared through CDC’s NSSP platform (*3*). The 10 jurisdictions included in this analysis reported data on a minimum of 75% of ED visits occurring within their jurisdictions, including a minimum of 90% of visits from Level 1–3 trauma centers.^§§^ Initial firearm injury encounters (including those classified as unintentional, intentional self-harm, assault, legal intervention, terrorism, and undetermined intent) were identified using a syndrome definition including diagnosis codes and chief complaint text fields (Supplementary Box, https://stacks.cdc.gov/view/cdc/118752).

Data on county-level social vulnerability were obtained from the 2018 SVI, which uses U.S. Census Bureau American Community Survey 2014–2018 5-year data^¶¶^ estimates for 15 population-based county-level sociodemographic indicators to form an overall social vulnerability metric, as well as four additional focused metrics representing themes of socioeconomic status, household composition and disability, racial and ethnic minority status and language proficiency, and housing type and transportation. The SVI includes ranked scores ranging from 0–1 applied to 3,142 counties in the United States.

Counties in the 10 FASTER-funded jurisdictions were categorized into tertiles (low, medium, high) of social vulnerability for the overall SVI, the SVI themes, and the individual indicators of each SVI theme, with higher values representing higher levels of social vulnerability. Crude rates of firearm injury ED visits (number of ED visits for firearm injuries per 100,000 ED visits) were calculated for each level of social vulnerability. A total of 647 (99.2%) of 652 counties sharing data with NSSP in the 10 jurisdictions had data on ED visits and the SVI, and were included in the analyses. Negative binomial regression models including fixed effects for jurisdictions were fit to estimate RRs and associated 95% CIs comparing rates among high and medium social vulnerability counties with those in low social vulnerability counties across the overall SVI, separate SVI themes, and individual SVI indicators.*** Regression analyses were conducted using SAS software (version 9.4; SAS Institute). SVI tertiles were included in the models as categorical variables. This activity was reviewed by CDC and was conducted consistent with applicable federal law and CDC policy.^†††^

During 2018–2021, the overall crude firearm injury ED visit rate among the 10 jurisdictions was 74 per 100,000 ED visits, with low, medium, and high social vulnerability counties experiencing rates of 55, 77, and 92 firearm injury ED visits per 100,000 ED visits, respectively. Compared with counties with low overall social vulnerability, rates of firearm injury ED visits were 1.34 and 1.80 times as high in counties with medium and high overall social vulnerability, respectively (Table). Similar patterns were observed for the SVI theme of socioeconomic status, with rates of firearm injury ED visits higher among counties with medium (RR = 1.27) and high (RR = 1.61) vulnerability compared with counties with low social vulnerability. This pattern was apparent for all four indicators of socioeconomic status, with the most pronounced differences in firearm injury ED visit rates observed when comparing SVI tertiles across the poverty indicator.

**TABLE:** Rates of ED visits for firearm injuries* in medium and high social vulnerability areas compared with rates in low social vulnerability areas^†^ — FASTER program, 10 U.S. jurisdictions,^§^ 2018–2021

SVI themes	SVI tertile (vulnerability level), RR^¶^ (95% CI)		
	1 (Low)**	2 (Medium)	3 (High)
**Overall**	**Ref**	**1.34 (1.22–1.47)**	**1.80 (1.50–2.16)**
**Socioeconomic status**	Ref	1.27 (1.15–1.39)	1.61 (1.33–1.94)
Percentage of persons living below poverty	Ref	1.40 (1.27–1.53)	1.95 (1.62–2.34)
Percentage of persons unemployed	Ref	1.18 (1.07–1.30)	1.39 (1.15–1.68)
Per capita income^††^	Ref	1.15 (1.04–1.26)	1.31 (1.09–1.59)
Percentage of persons aged ≥25 yrs with no HS diploma	Ref	1.20 (1.08–1.32)	1.43 (1.18–1.74)
**Household composition and disability status**	Ref	1.02 (0.93–1.12)	1.05 (0.86–1.26)
Percentage of persons aged ≥65 yrs	Ref	0.89 (0.81–0.98)	0.80 (0.66–0.97)
Percentage of persons aged <18 yrs	Ref	1.07 (0.97–1.18)	1.14 (0.94–1.39)
Percentage of persons living with a disability	Ref	1.10 (0.99–1.21)	1.20 (0.99–1.46)
Percentage of households with single parents and children	Ref	1.18 (1.07–1.29)	1.39 (1.15–1.67)
**Racial and ethnic minority status and language proficiency**	Ref	1.08 (0.97–1.20)	1.17 (0.94–1.44)
Percentage of racial and ethnic minority residents	Ref	1.25 (1.13–1.37)	1.55 (1.28–1.89)
Percentage of persons with limited English proficiency	Ref	0.97 (0.88–1.08)	0.94 (0.77–1.16)
**Housing type and transportation**	Ref	1.32 (1.21–1.45)	1.75 (1.45–2.11)
Percentage of housing structures with ≥10 units	Ref	1.00 (0.91–1.11)	1.00 (0.82–1.22)
Percentage of housing units that are mobile home units	Ref	0.92 (0.83–1.01)	0.84 (0.70–1.03)
Percentage of households with more persons than rooms	Ref	1.06 (0.96–1.17)	1.12 (0.92–1.37)
Percentage of households with no vehicle access	Ref	1.10 (1.01–1.20)	1.21 (1.01–1.45)
Percentage of persons living in group quarters	Ref	1.20 (1.09–1.32)	1.45 (1.20–1.75)

**Abbreviations:** ATSDR = Agency for Toxic Substances and Disease Registry; ED = emergency department; FASTER = Firearm Injury Surveillance Through Emergency Rooms; HS = high school; NSSP = National Syndromic Surveillance Program; Ref = referent group; RR = rate ratio; SVI = social vulnerability index.

* Defined using CDC’s syndrome definition based on a combination of discharge diagnosis codes and chief complaint terms identifying initial encounters for a firearm injury, including those classified as unintentional, intentional self-harm, assault, legal intervention, terrorism, and undetermined intent.

^†^ County-level social vulnerability data were obtained from the 2018 CDC/ATSDR SVI. Counties were categorized into groups (low, medium, high) of social vulnerability for the overall SVI, its four themes, and the individual indicators comprising each SVI theme based on tertile distributions across the counties. Higher values of the overall SVI, SVI themes, and SVI indicators represent greater levels of social vulnerability.

^§^ The 10 FASTER-funded jurisdictions were District of Columbia, Florida, Georgia, New Mexico, North Carolina, Oregon, Utah, Virginia, Washington, and West Virginia. Data from these jurisdictions were shared with CDC’s NSSP (accessed March 16, 2022). Among 652 counties with facilities sharing data with NSSP in the 10 jurisdictions, 647 (99%) had data on the SVI and at least one ED visit and were included in rate calculations.

^¶^ Rates of firearm injury ED visits (number of ED visits for firearm injuries per 100,000 ED visits) for each level of county social vulnerability were calculated. Negative binomial regression models including fixed effects for jurisdictions were fit to estimate RRs and associated 95% CIs comparing rates among high and medium social vulnerability counties with those in low social vulnerability counties across the overall SVI, separate SVI themes, and individual SVI indicators.

** Referent group was low social vulnerability areas.

^††^ Per capita income was reverse-coded.

For the housing type and transportation theme, rates of firearm injury ED visits were also higher among medium (RR = 1.32) and high (RR = 1.75) social vulnerability counties compared with low social vulnerability counties. This pattern was apparent for two of five indicators constituting the theme: percentage of persons living in group quarters and percentage of households with no vehicle access.

Although ED visit rates were not higher in medium and high social vulnerability counties for the two SVI themes of household composition and disability status and racial and ethnic minority status and language proficiency, among specific indicators for each, rates were higher among counties with higher percentages of single-parent households and persons identifying as a racial or ethnic minority; rates were lower among counties with higher percentages of persons aged ≥65 years.

## Discussion

In this multistate report analyzing syndromic surveillance ED data for firearm injuries across FASTER-funded jurisdictions, counties with higher overall social vulnerability experienced higher rates of firearm injury ED visits during 2018–2021. Higher community social vulnerability has been previously associated with higher rates of firearm deaths (*4*). The findings of this report indicate that social vulnerability is also associated with the percentage of ED visits that are for firearm injuries.

In this analysis, among all ED visits, higher proportions of firearm injury ED visits occurred in low socioeconomic status communities. An index of neighborhood disadvantage, including poverty and unemployment, has been previously associated with higher numbers of firearm injuries (*5*), and surrounding poverty and higher income inequality have been linked to higher firearm homicide rates (*6*,*7*). In the present analysis, rates of firearm injury visits were also associated with additional indicators, including proportion of racial and ethnic minority persons. Current and historical inequities that marginalize some racial and ethnic minority groups in the United States might contribute to elevated rates of firearm injuries in these communities (*8*). For example, structural racism, in the form of redlining, a discriminatory practice of systematic disinvestment of neighborhoods and denial of service provision (including financial services) to residents of neighborhoods that include substantial numbers of racial and ethnic minority and low-income residents, has been associated with higher rates of firearm injuries in an urban setting (*9*); evidence indicates that racial residential segregation has also been predictive of racial disparities in firearm-related homicides (*10*). Patterns of firearm injury visit rates also varied by age, vehicle accessibility, housing density, and single-parent household status. Together with additional context and understanding of historical and structural factors affecting specific communities, these data can help guide tailored prevention efforts and partnerships to reduce inequities in risk for firearm injuries.

The findings in this report are subject to at least five limitations. First, data are limited to 10 U.S. jurisdictions and are not nationally representative. Second, the syndrome definition used in this study to identify firearm injury ED visits does not distinguish the injury intent; the distribution of firearm injuries across levels of social vulnerability might be different for specific intents. In addition, the definition might under- or overestimate ED visits related to firearm injuries because of possible variation in coding practices and reporting of visit-level data across facilities. Third, the number of facilities sharing data with NSSP can vary over time. Potential fluctuations in facility participation were accounted for by calculating a rate indicating the proportion of the total number of ED visits for firearm injuries. However, rates, and thereby, comparisons, across SVI tertiles could be influenced by changes in the denominator or characteristics of the populations usually served by participating facilities. Fourth, the smallest geographic level at which these firearm injury data were available at the time of this report is at the county level, which limits the ability to examine the distribution of firearm injuries across smaller geographic levels (e.g., census tract). Finally, the SVI is based on 5-year estimates during 2014–2018, and ED visits during 2018–2021 were analyzed. The timing of the data and ecological design limit the ability to draw causal conclusions and examine current or historical determinants of firearm injuries.

Timelier ED data can help health departments and clinical and community partners collaboratively identify communities disproportionately experiencing firearm injuries. SVI data can help focus prevention efforts on reducing and addressing the effects of the underlying drivers of inequities using strategies with the best available evidence, including creating protective community environments, strengthening economic supports, and intervening to reduce harms and prevent future risk (e.g., with hospital-based violence intervention programs) (*1*,*2*).

**Summary**

**What is already known about this topic?**

At least 100,000 persons in the United States experience a fatal or nonfatal firearm injury each year.

**What is added by this report?**

During 2018–2021, among 10 jurisdictions participating in CDC’s Firearm Injury Surveillance Through Emergency Rooms program, counties with higher overall social vulnerability experienced higher proportions of emergency department visits for firearm injuries.

**What are the implications for public health practice?**

Monitoring firearm injury emergency department visits by county-level social vulnerability can help guide tailored prevention efforts that address inequities in social and structural conditions that contribute to risk for violence, including creating protective community environments, strengthening economic supports, and intervening to reduce harms and prevent future risk.

## Footnotes

*
https://www.cdc.gov/injury/wisqars/index.html

†
https://www.cdc.gov/violenceprevention/firearms/funded-surveillance.html

§ Analyses were limited to ED encounters. NSSP is a collaboration among CDC; local and state health departments; and federal, academic, and private sector partners. Electronic patient encounter data are collected from EDs, urgent and ambulatory care centers, inpatient health care settings, and laboratories. https://www.cdc.gov/nssp/index.html

¶ The 10 FASTER-funded jurisdictions were the District of Columbia, Florida, Georgia, New Mexico, North Carolina, Oregon, Utah, Virginia, Washington, and West Virginia.

**
https://www.atsdr.cdc.gov/placeandhealth/svi/index.html

††
https://www.norc.org/PDFs/A%20Blueprint%20for%20U.S.%20Firearms%20Data%20Infrastructure/Improving%20Data%20Infrastructure%20to%20Reduce%20Firearms%20Violence_Final%20Report.pdf

§§
https://www.amtrauma.org/page/traumalevels

¶¶
https://www.census.gov/programs-surveys/acs/technical-documentation/table-and-geography-changes/2018/5-year.html

*** The number of facilities sharing data with NSSP can vary over time and potentially influence ED visit trends. Although this study used a cross-sectional analysis using data aggregated across years and did not examine trends, sensitivity analyses were conducted by restricting to facilities consistently reporting informative data (specifically, facilities with a coefficient of variation ≤40 and average number of weekly discharge diagnoses with useful information of ≥75 through the analysis period; average number of weekly discharge diagnoses with useful information was not applied to facilities in the District of Columbia). Results from the sensitivity analyses were similar to results presented in this report, which analyzed data from all facilities sharing data with NSSP in the 10 FASTER-funded jurisdictions during the study period.

††† 45 C.F.R. part 46.102(l)(2), 21 C.F.R. part 56; 42 U.S.C. Sect. 241(d); 5 U.S.C. Sect. 552a; 44 U.S.C. Sect. 3501 et seq.

## References

[1] David-Ferdon C, Vivolo-Kantor AM, Dahlberg LL, Marshall KJ, Rainford N, Hall JE. A comprehensive technical package for the prevention of youth violence and associated risk behaviors. Atlanta, GA: US Department of Health and Human Services, CDC; 2016. https://www.cdc.gov/violenceprevention/pdf/yv-technicalpackage.pdf

[2] Stone DM, Holland KM, Bartholow B, Crosby AE, Davis S, Wilkins N. Preventing suicide: a technical package of policies, programs, and practices. Atlanta, GA: US Department of Health and Human Services, CDC; 2017. https://www.cdc.gov/violenceprevention/pdf/suicidetechnicalpackage.pdf

[3] Zwald ML, Holland KM, Bowen DA, Using the Centers for Disease Control and Prevention’s National Syndromic Surveillance Program data to monitor trends in US emergency department visits for firearm injuries, 2018 to 2019. Ann Emerg Med 2022;79:465–73. 10.1016/j.annemergmed.2022.01.016 35277293 PMC9299533

[4] Phelos HM, Deeb AP, Brown JB. Can social vulnerability indices predict county trauma fatality rates? J Trauma Acute Care Surg 2021;91:399–405. 10.1097/TA.0000000000003228 33852559 PMC8375410

[5] Dalve K, Gause E, Mills B, Floyd AS, Rivara FP, Rowhani-Rahbar A. Neighborhood disadvantage and firearm injury: does shooting location matter? Inj Epidemiol 2021;8:10. 10.1186/s40621-021-00304-2 33678193 PMC7938602

[6] Kegler SR, Dahlberg LL, Vivolo-Kantor AM. A descriptive exploration of the geographic and sociodemographic concentration of firearm homicide in the United States, 2004-2018. Prev Med 2021;153:106767. 10.1016/j.ypmed.2021.106767 34416223 PMC8667260

[7] Rowhani-Rahbar A, Quistberg DA, Morgan ER, Hajat A, Rivara FP. Income inequality and firearm homicide in the US: a county-level cohort study. Inj Prev 2019;25(Suppl 1):i25–30. 10.1136/injuryprev-2018-043080 30782593

[8] Nation M, Chapman DA, Edmonds T, Social and structural determinants of health and youth violence: shifting the paradigm of youth violence prevention. Am J Public Health 2021;111(S1):S28–31. 10.2105/AJPH.2021.306234 34038155 PMC8157807

[9] Jacoby SF, Dong B, Beard JH, Wiebe DJ, Morrison CN. The enduring impact of historical and structural racism on urban violence in Philadelphia. Soc Sci Med 2018;199:87–95. 10.1016/j.socscimed.2017.05.038 28579093 PMC7437144

[10] Wong B, Bernstein S, Jay J, Siegel M. Differences in racial disparities in firearm homicide across cities: the role of racial residential segregation and gaps in structural disadvantage. J Natl Med Assoc 2020;112:518–30. 10.1016/j.jnma.2020.05.014 32641258

